# Integrative Computational and Experimental Approaches Reveal the Protective Role of Moderate Caffeine Intake Against Apical Periodontitis Induced Bone Loss

**DOI:** 10.1111/iej.70105

**Published:** 2026-02-08

**Authors:** Matheus Ferreira Lima Rodrigues, Deborah Ribeiro Frazão, Deiweson Souza‐Monteiro, Vinicius Ruan Neves dos Santos, Felipe Oliveira Nunes, João Daniel Mendonça de Moura, Thamires Campos Gomes, Jorddy Neves Cruz, Cristiane do Socorro Ferraz Maia, Rodrigo A. Cunha, Fabrício Mezzomo Collares, Rogerio de Castilho Jacinto, Rafael Rodrigues Lima

**Affiliations:** ^1^ Laboratory of Functional and Structural Biology, Institute of Biological Sciences Federal University of Pará Belém Pará Brazil; ^2^ Laboratory of Pharmacology of Inflammation and Behaviour, Faculty of Pharmacy, Institute of Health Science Federal University of Pará Belém Pará Brazil; ^3^ iCBR‐Institute for Clinical and Biomedical Research, Faculty of Medicine University of Coimbra Coimbra Portugal; ^4^ Department of Dental Materials, School of Dentistry Federal University of Rio Grande Do Sul Porto Alegre Rio Grande Do Sul Brazil; ^5^ Department of Preventive and Restorative Dentistry, Endodontic Section, School of Dentistry São Paulo State University (UNESP) Araçatuba São Paulo Brazil

**Keywords:** alveolar bone, antioxidant, apical periodontitis, bone loss, caffeine, inflammation, oxidative stress

## Abstract

**Aim:**

To investigate whether moderate systemic caffeine intake modulates the progression of apical periodontitis (AP) and associated alveolar bone loss, combining in vivo rat experiments with *in silico* molecular docking to explore potential mechanisms.

**Methodology:**

Male Wistar rats were randomly assigned to four groups (*n* = 8 per group): control, caffeine, AP, AP + caffeine. AP was induced by pulp exposure of mandibular first molars and allowed to develop for 28 days. Animals in caffeine groups received 10 mg/kg/day by orogastric gavage during the experimental period. The antioxidant capacity of caffeine was assessed by DPPH• and ABTS• + assays. Systemic oxidative status was evaluated by blood reduced glutathione (GSH) and thiobarbituric acid reactive substances (TBARS). Histology, Picro‐Sirius red staining for collagen, and micro‐computed tomography (micro‐CT) analysis of alveolar bone (BV/TV, Tb.N, Tb.Sp, porosity, lesion volume) were performed. Molecular docking against adenosine A_1_ and A_2__A_ receptors was used to probe caffeine–receptor interactions.

**Results:**

Caffeine showed relevant radical‐scavenging activity in vitro (DPPH• assay). AP induced systemic redox imbalance, marked inflammatory infiltration, collagen loss and increased lesion volume. Moderate caffeine intake restored redox markers (↑GSH, ↓TBARS), attenuated inflammatory infiltrate, preserved collagen content and reduced lesion volume (AP + caffeine vs. AP; *p* < 0.05). Micro‐CT demonstrated improved alveolar bone microarchitecture in AP + caffeine group (increased BV/TV and Tb.N; reduced Tb.Sp and porosity). Molecular docking indicated stable hydrophobic and hydrogen‐bond interactions of caffeine within A_1_ and A_2__A_ receptor binding pockets, supporting an antagonistic effect on adenosine signalling consistent with reduced pro‐inflammatory activation.

**Conclusion:**

Moderate systemic caffeine (10 mg/kg/day) attenuates apical periodontitis progression and preserves alveolar bone quality in rats, associated with antioxidant activity and a probable modulation of adenosine receptor‐mediated inflammatory pathways.

## 1 Introduction

Apical periodontitis (AP) is a chronic inflammatory condition affecting the periradicular tissues, primarily resulting from persistent microbial infection within the root canal system (Frazão et al. 2023). Upon pulp infection, there is a rapid recruitment of polymorphonuclear leukocytes and monocytes to the infected site, leading to local activation of innate and adaptive pathways, osteoclastic bone resorption, and a marked increase in the production of reactive oxygen species (ROS). The resulting oxidative stress environment not only exacerbates tissue damage but also sustains the inflammatory cascade, contributing to disease progression (Dal‐Fabbro, Cosme‐Silva, Capalbo, et al. 2021; Frazão et al. 2023; Liapatas et al. 2003; Stashenko et al. 1998). Within this pathophysiological framework, therapeutic approaches aimed at reestablishing inflammatory and redox homeostasis are of considerable interest.

The gold standard treatment for AP is conventional endodontic therapy, which aims to eliminate the intraradicular infection through mechanical instrumentation, chemical irrigation with antimicrobial solutions, and three‐dimensional obturation of the root canal system, thus creating favourable conditions for periapical tissue repair (Pirani and Camilleri 2023). While the success rate of root canal treatment is generally high, periapical healing may be delayed or impaired in cases where there is persistent oxidative stress and an exacerbated inflammatory response (Holland et al. 2017).

Within this context, therapeutic strategies capable of restoring both inflammatory and redox homeostasis are of considerable interest. Among the strategies explored to counteract inflammation and oxidative stress, caffeine has gained increasing attention. Caffeine (1,3,7‐trimethylxanthine) is the most widely consumed psychoactive substance worldwide, primarily due to its natural presence in commonly ingested products such as coffee, tea, cocoa, and energy supplements (Ahluwalia and Herrick 2015). Caffeine possesses significant anti‐inflammatory and antioxidant activities (Ősz et al. 2022), which may be particularly relevant in chronic inflammatory and oxidative stress‐driven pathologies.

Mechanistically, moderate doses of caffeine act as a non‐selective antagonist of adenosine receptors (IJzerman et al. 2022), modulating intracellular cyclic AMP levels and downstream redox‐sensitive signalling pathways, mainly through A_2A_ receptors (López‐Cruz et al. 2018; Cunha 2019). This antagonism is linked to enhanced endogenous antioxidant defences and reduced production of ROS in various experimental models (Leite et al. 2011; Khan et al. 2019). In addition, adenosine receptors critically control inflammation (Haskó and Pacher 2008) and, in particular, A_2A_ receptor overfunction increases interleukin‐6 signalling by human gingival fibroblasts, which plays a pivotal role in the immunomodulatory process in periodontal disease (Murakami et al. 2000) and promoted metabolically active intracellular *P. gingivalis* in gingival epithelial cells that are closely associated with periodontal disease (Spooner et al. 2014).

This anticipated beneficial effect of caffeine to counteract periodontal lesions involving inflammation and redox imbalance is in surprising contrast with animal studies, which mostly concluded that caffeine actually aggravates experimental periodontitis (Bezerra et al. 2013) and apical periodontitis (Dal‐Fabbro, Cosme‐Silva, de Rezen Silva Martins Oliveira, et al. 2021). Importantly, these two studies tested abnormally high exposures to caffeine, far exceeding the average human intake of caffeine. The tested dose of caffeine is of uppermost importance since caffeine has bell‐like effects on several physiological parameters, as best heralded by the opposite effects of high doses of caffeine bolstering inflammation and bone demineralization (Dal‐Fabbro, Cosme‐Silva, Capalbo, et al. 2021; Shin et al. 2015) in contrast to moderate doses of caffeine that dampen inflammation and protect against bone loss (Horrigan et al. 2006; Folwarczna et al. 2013). This might also apply to oral health and teeth preservation since, when tested at doses equivalent to average human consumption, we previously reported that caffeine intake decreases alveolar bone damage induced by binge‐like ethanol consumption in adolescent female rats (Maia et al. 2020).

Given this dose‐dependent duality, a deeper understanding of caffeine's impact on alveolar bone is crucial, particularly to evaluate its potential as an adjuvant therapeutic agent in inflammatory oral diseases such as AP. In this context, we hypothesized that, at physiologically relevant doses, caffeine would exert antioxidant and anti‐inflammatory effects, reducing periapical tissue damage. Thus, the present study aimed to investigate the effects of systemically administered moderate‐dose caffeine in a rat model of AP, combining in vivo experimentation with *in silico* computational analyses to provide a comprehensive mechanistic perspective.

## 2 Materials and Methods

### 2.1 Molecular Docking

**FIGURE 1:**
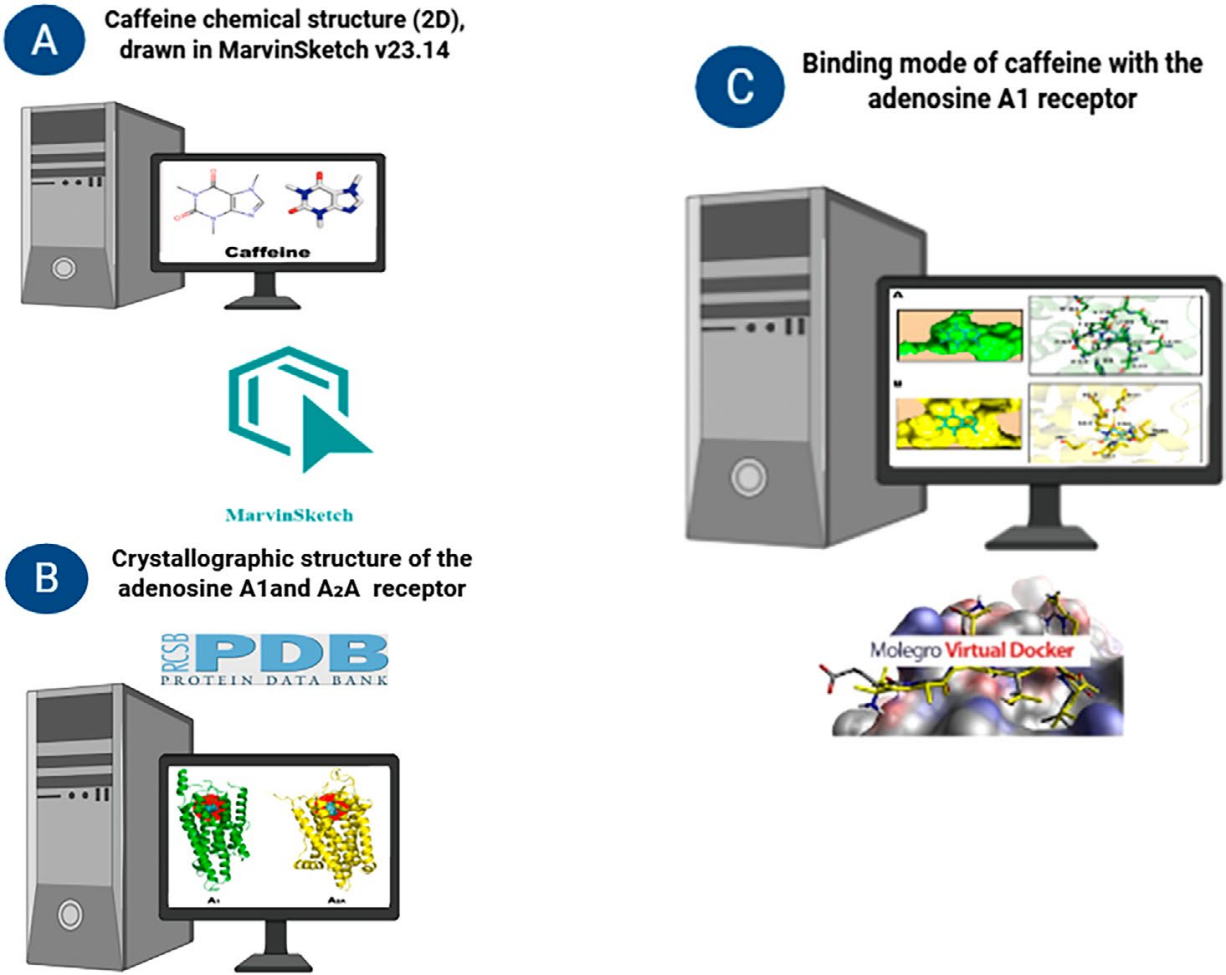
Schematic representation of the molecular modelling approaches and structures used in this study. (A) Two‐dimensional chemical structure of caffeine, drawn using MarvinSketch v23.14. (B) Crystal structures of the adenosine A_1_ and A_2__A_ receptors, obtained from the Protein Data Bank (PDB). (C) Binding mode of caffeine with the adenosine A1 receptor, determined using Molegro Virtual Docker software.

We employed molecular modelling approaches to evaluate the interaction of caffeine with adenosine A_1_ and A_2A_ receptors, which are involved in the modulation of inflammatory processes and osteometabolism (Figure 1) (Mediero and Cronstein 2013; Haskó and Pacher 2008). The molecular structure of caffeine was constructed using MarvinSketch v.23.14 and subsequently optimised at the B3LYP/6‐31G level of theory (Becke 1993) using Gaussian 16. The crystallographic structure of the adenosine A1 receptor was retrieved from the Protein Data Bank (PDB ID: 5N2S) (Cheng et al. 2017), while the structure of the A_2__A_ receptor was obtained from PDB ID: 3RFM (Doré et al. 2011).

To validate the docking protocol, a redocking procedure was performed using the crystallographic ligand present in the A_1_ receptor structure (PDB ID: 5N2S). This step aimed to confirm the reproducibility of the method and its accuracy in predicting the experimental conformation of the ligand within the orthosteric site. Redocking was conducted using Molegro Virtual Docker 5.5 (MVD 5.5) (Thomsen and Christensen 2006) by removing the co‐crystallised ligand and re‐docking it into the same binding pocket. The search space was defined to fully encompass the original ligand‐binding cavity.

The MolDock Score (GRID) function was applied with a grid resolution of 0.30 Å and a search radius of 7 Å, covering the entire orthosteric binding site. The MolDock SE algorithm was used with the following parameters: 50 independent runs, 3000 maximum interactions, a population size of 200, 300 evaluation steps, a neighbour distance factor of 1.0, and an energy threshold of 100. After docking, the lowest‐energy pose (most negative MolDock Score) was compared with the crystallographic conformation, and the Root Mean Square Deviation (RMSD) was calculated to assess the accuracy of the protocol. RMSD values below 2.0 Å were considered indicative of a reliable and reproducible method (Forli et al. 2016; Santos et al. 2021; Costa et al. 2020).

The same validated docking protocol was then applied to the caffeine molecule using the A_1_ receptor. This procedure was not extended to the A_2A_ receptor, as its crystallographic structure already contains caffeine co‐crystallised within the binding pocket (Doré et al. 2011).

### 2.2 Caffeine Antioxidant Activity

The ABTS^•+^ (2,2′‐Azino‐bis‐3‐ethylbenzothiazoline‐6‐sulfonic acid) and DPPH^•^ (2,2‐diphenyl‐1‐picrylhydrazyl) assays were methods used to assess the antioxidant capacities of caffeine. The antioxidant potential of the studied compound was determined according to its equivalence to the potent antioxidant Trolox (6‐hydroxy‐2,5,7,8‐tetramethylchromono‐2‐carboxylic acid) and a water‐soluble synthetic vitamin E analog.

The ABTS^•+^ radical scavenging assay was determined according to the methodology adapted from Miller et al. (1993), and modified by Re et al. (1999), ABTS• + was prepared using 7 mM ABTS•+, and 140 mM of potassium persulfate incubated at room temperature without light for 16 h. Then, the solution was diluted with phosphate‐buffered saline until it reached an absorbance of 0.700 (± 0.02) at 734 nm.

The synthetic antioxidant Trolox was used as a standard solution for the calibration curve. To measure the antioxidant capacity, 2.97 mL of the ABTS• + solution was transferred to a cuvette and the absorbance at 734 nm was determined using a Nm Kasvi spectrophotometer. Then, 0.03 mL of the sample was added and after 5 min, the second reading was performed. The results were expressed as mM. The values found for the samples were compared to the Trolox standard (1 mM).

The DPPH analysis was performed to analyse the potential of caffeine to dampen the levels of the 1,1‐diphenyl‐2‐picrylhydrazyl (DPPH•) radical, a violet chromophore, resulting in the formation of the hydrogenated DPPH product, which is yellow or colourless. The test was carried out according to the method proposed by Blois (Blois 1958). To measure the antioxidant capacity, we first determined the absorbance of the 0.1 mM DPPH• solution (2,2‐diphenyl‐1‐picrylhydrazyl) in ethanol. Subsequently, 0.6 mL of DPPH• solution, 0.35 mL of distilled water, and 0.05 mL of the sample were mixed and placed in a water bath at 37°C for 30 min. After that, the absorbances were determined in a Nm Kasvi spectrophotometer at 517 nm. The synthetic antioxidant Trolox was used as a standard solution for the calibration curve. The results were expressed as mM. The values found for the samples were compared to the Trolox standard (1 mM).

### 2.3 Ethics Statement and Experimental Groups

**FIGURE 2:**
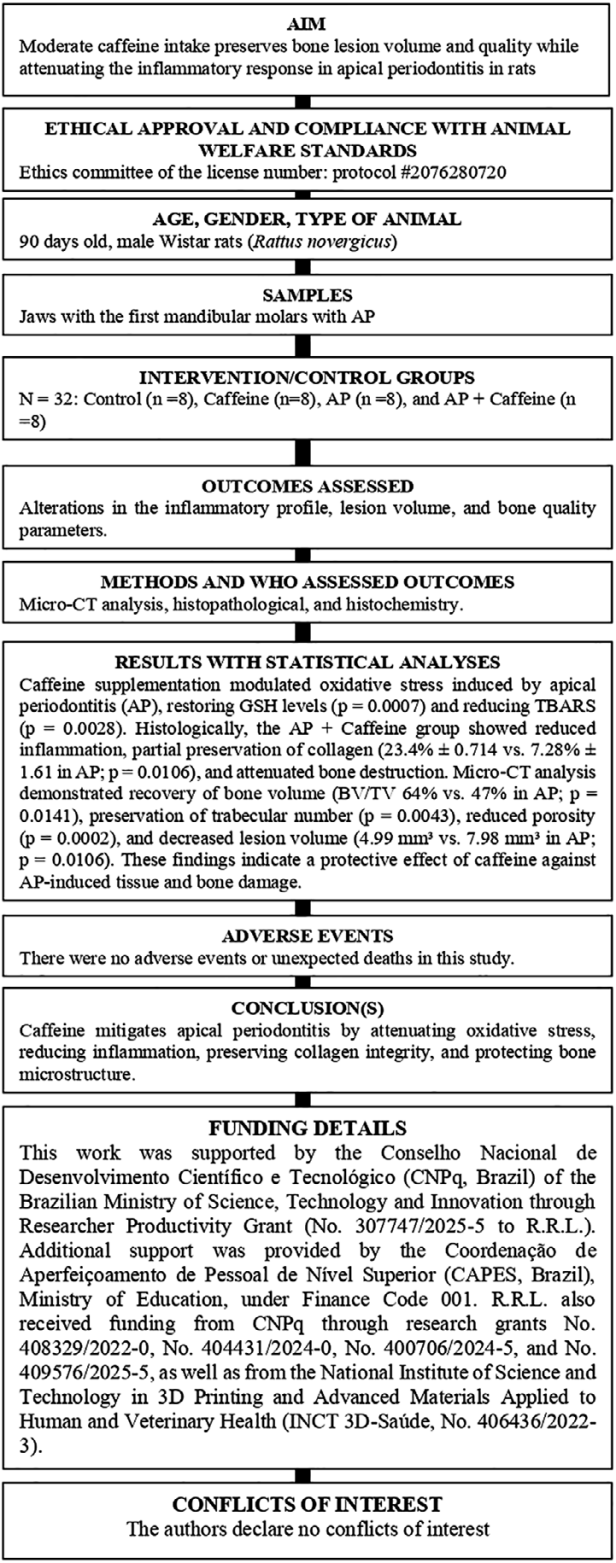
PRIASE 2021 flowchart.

This study was approved by the Animal Research Ethics Committee (number 2076280720). The study followed the ARRIVE 2.0 guidelines (du Sert et al. 2020), the NIH Guide for the Care and Use of Laboratory Animals (National Research Council (US) Committee for the Update of the Guide for the Care and Use of Laboratory Animals 2011), and the Preferred Reporting Items for Animal Studies in Endodontology (PRIASE) 2021 guidelines (Nagendrababu et al. 2021), including the PRIASE flowchart (Figure 2).

Thirty‐two male Wistar rats ( *Rattus norvegicus* ), approximately 90 days old and weighing between 175 and 250 g, were randomly housed in polypropylene cages measuring 30 × 20 × 12 cm, with no more than four rats per cage to prevent overcrowding. The animals had unrestricted access to food and water and were kept under a consistent 12‐h light/dark cycle. Room conditions were carefully regulated, maintaining a stable temperature of 25°C ± 1°C and a relative humidity of 50% ± 10%. Bedding materials (wood shavings) were changed weekly to ensure proper hygiene.

The animals were randomised (using https://www.randomizer.org/ software) into four experimental groups (*n* = 8/group): (I) control group, which included animals not subjected to lesion induction; (II) Caffeine group, consisting of animals not subjected to lesion induction with supplementation of caffeine; (III) AP group, consisting of animals that underwent access surgery without supplementation; and (IV) apical periodontitis + caffeine (AP + Caffeine) group, comprising animals that underwent lesion induction surgery and received caffeine supplementation. As eligibility requirements, we evaluated the absence of evident health issues during veterinary checks, consistent weight gain throughout the acclimation period, and successful induction of AP (verified by the standardised pulp exposure protocol in the AP groups). Animals in the control group retained healthy periapical conditions for the duration of the study. No exclusions were made since all subjects fulfilled health standards, and every surgical procedure was carried out without complications.

### 2.4 Apical Periodontitis Induction

Experimental induction of AP was carried out based on the studies of Aksoy et al. (2019), Frazão et al. (2023), and Matos‐Sousa et al. (2024). We anaesthetised the animals from the AP and AP + Caffeine groups intraperitoneally using a combination of 2% xylazine (8 mg/kg) and 10% ketamine (90 mg/kg) and positioned them on an operating table. A #1/4 carbide burr (KaVo Dental, Biberach an der Riß, Germany) was utilised in conjunction with the X‐Smart Plus motor (Dentsply Maillefer, Ballaigues, Switzerland) to perform pulp exposure. The motor was calibrated to a rotational speed of 1200 rpm with a torque setting of 4.0 Newton‐centimetres. This methodology enabled the exposure of the pulpal chamber in both the left and right first lower molars. Pulpal bleeding confirmed root canal access. The exposed teeth remained open to the oral environment for 28 days to facilitate the development of apical lesions. To mitigate pain and discomfort, we administered dipyrone subcutaneously at a dose of 100 mg/kg daily for 3 days.

### 2.5 Caffeine Administration and Sample Collection

Twenty‐four hours after lesion induction, caffeine (Sigma‐Aldrich) was administered via orogastric gavage to the caffeine and AP + caffeine groups at a dose of 10 mg/kg/day for 28 days. For the orogastric gavage, a stainless‐steel cannula was used to ensure safe administration. The procedure was executed by experienced technicians, and daily monitoring confirmed that no oral or esophageal injuries were present in any of the animals during the treatment. The dose of caffeine was selected based on the study by Maia et al. (2020), which demonstrated efficacy in preserving alveolar bone microarchitecture and confirmed antioxidant properties at this dose without adverse effects. Dose adjustments were made based on weekly body weight measurements. Furthermore, this dose models a moderate exposure to caffeine on the basis that 10 mg/kg in rats is roughly equivalent to a dose of 250 mg of caffeine in humans representing 2–3 cups of coffee, using the metabolic body weight correction (Gilbert 1976; Fredholm et al. 1999).

During the same period, control and AP groups received distilled water via the same administration route. Throughout the study, animals were monitored twice daily for pain indicators (including reduced mobility and altered grooming patterns) and surgical site infections. Furthermore, body weight was recorded weekly to assess overall health status. No animals exhibited adverse events exceeding predefined humane endpoint criteria (> 20% weight loss, persistent anorexia, or severe lethargy), and none required early euthanasia.

**FIGURE 3:**
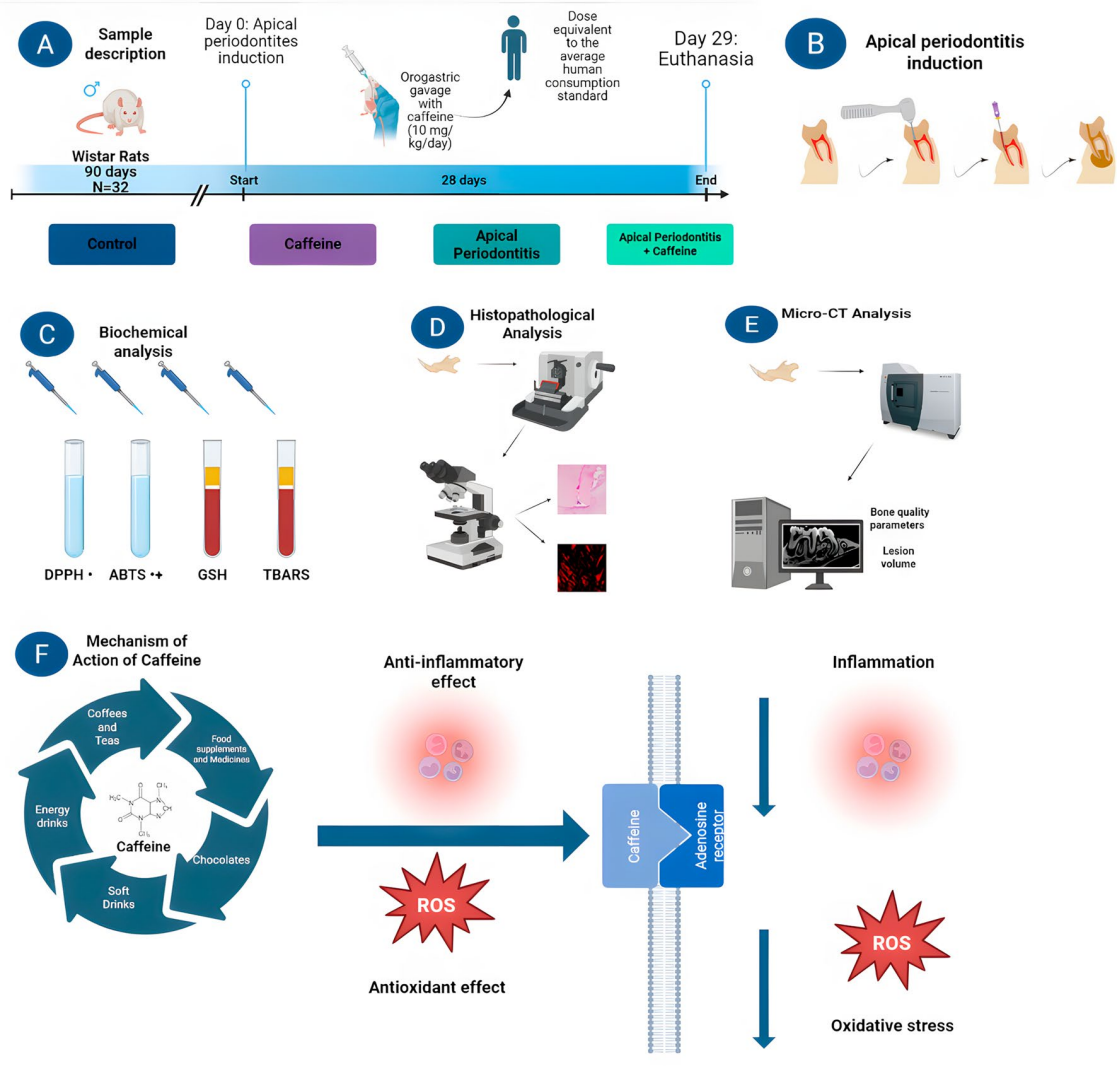
Experimental design and mechanism of action of caffeine. Panels (A–E) illustrate the sample description and experimental timeline. Apical periodontitis was induced in Wistar rats (*n* = 32) at Day 0, followed by 28 days of orogastric gavage with caffeine (10 mg/kg/day), a dose equivalent to average human consumption. Euthanasia was performed on Day 29, and samples were subjected to biochemical, histopathological, and micro‐CT analyses, including assessments of oxidative stress markers (DPPH, ABTS, GSH, TBARS) and bone quality parameters and lesion volume. Panel (F) depicts the proposed mechanism of action of caffeine, present in energy drinks, coffees, teas, food supplements, and medicines, highlighting its anti‐inflammatory and antioxidant effects in mitigating inflammation and oxidative stress.

All procedures were conducted under the supervision of qualified personnel with certified training in laboratory animal welfare, thus ensuring strict adherence to ethical standards. Twenty‐four hours after administering the last dose of caffeine, animals were euthanized using ketamine (90 mg/kg) and xylazine (10 mg/kg) for blood collection and subsequent biochemical analysis. Mandibles were harvested and processed for various examinations as outlined in Figure 3, which summarises the complete experimental workflow.

### 2.6 Oxidative Biochemical Assays

To evaluate caffeine's antioxidant properties and its potential to mitigate AP‐induced damage, we analysed plasma markers of oxidative status. Blood samples were collected in tubes containing 50 μL of 5% ethylenediaminetetraacetic acid (EDTA) and centrifuged at 3000 rpm for 10 min. Plasma was separated, aliquoted into Eppendorf tubes, and stored at −80°C for subsequent analysis of reduced glutathione (GSH) levels and thiobarbituric acid reactive substances (TBARS).

GSH levels were measured using a modified Ellman method (Ellman 1959). Briefly, plasma samples were incubated with 5,5‐dithiobis‐2‐nitrobenzoic acid (DTNB), which reacts with sulfhydryl groups in GSH to form 2‐nitro‐5‐thiobenzoic acid (TNB). Absorbance was measured immediately (T0) and 3 min after DTNB addition (T3) at 412 nm using a spectrophotometer. The difference between T3 and T0 absorbance values was proportional to GSH concentration. Results were expressed as μmol/mL.

Lipid peroxidation was evaluated by quantifying TBARS, as previously described (Kohn and Liversedge 1944; Percário 1994). Plasma samples were mixed with thiobarbituric acid (TBA) and heated at 94°C for 60 min to form a malondialdehyde (MDA)‐TBA adduct. After cooling, n‐butyl alcohol was added, and the mixture was vortexed and centrifuged at 2500 rpm for 10 min. The organic phase absorbance was measured at 535 nm, and MDA concentration was calculated using a molar extinction coefficient of 1.56 × 10^5^ M^−1^ cm^−1^. Results were expressed as nmol/mL.

### 2.7 Histopathological Analysis

One of the hemimandibles was post‐fixed in 4% formaldehyde for 24 h and subsequently demineralized in 10% EDTA for 90 days, with weekly solution changes until histological processing (Frazão et al. 2023). Once decalcification was complete, the tissues were rinsed in running water, dehydrated using a series of increasing ethanol concentrations (70%, 80%, 90%, Absolute I, and Absolute II), diaphanized in xylene, and embedded in paraffin (McCormick Scientific, McHenry, IL, USA). From the paraffin blocks, sections with a thickness of 5 μm were obtained using a microtome (Leica Microsystems, Nussloch, Germany). The sections were stained either with haematoxylin–eosin (HE) to evaluate tissue morphology and inflammatory profiles or with, PicroSirius Red to evaluate collagen content.

Bone preservation and inflammation were characterised using images captured from HE‐stained slides with a colour digital camera (DS‐Fi3, Nikon, Tokyo, Japan) attached to a microscope (Nikon Eclipse Ci‐S, Tokyo, Japan). The inflammatory profile of periapical lesions was evaluated in semi‐serial sections along the entire length of the mandible. The severity of the lesions was determined based on the intensity, characteristics, and extension of the inflammatory infiltration, as well as the preservation of the cementum and the integrity of the alveolar bone. The histopathological assessment was carried out by two independent, calibrated examiners who were blinded to the treatment groups.

PicroSirius Red‐stained sections of alveolar bone tissue were analysed under polarised light microscopy (40X optical lens) to evaluate collagen content (Junqueira et al. 1979; Matos‐Sousa et al. 2024). Images were captured using a microscope system equipped with polarising filters. Collagen area was quantified using ImageJ software by calculating the arithmetic mean from five fields/sections per sample, with results expressed as percentage area (Matos‐Sousa et al. 2024).

### 2.8 Computed X‐Ray Microtomography (Micro‐CT)

For microtomography evaluation, the other hemimandible was subjected to micro‐CT (MicroCT.SMX‐90 CT; Shimadzu Corp., Kyoto, Japan). Images were captured at a rotation angle of 360° at an intensity of 70 kV and 100 mA. Images were then reconstituted by using inspeXio SMX‐90CT software (Shimadzu Corp., Kyoto, Japan), with a voxel size of 10 μm and a resolution of 1024 × 1024, which resulted in 541 images per sample. All datasets were exported using the Joint Photographic Expert Group (JPEG) and Digital Imaging and Communications in Medicine (DICOM) files (Matos‐Sousa et al. 2024; Souza‐Monteiro et al. 2021).

The hemimandibles were then reconstructed using the CTAn software with JPEG datasets (V1.15.4.0; Bruker, Kontich, Belgium) to verify alveolar bone quality. The hemimandibles were placed in a standard position where the interradicular alveolar bone of the inferior first molar could be observed in the coronal view. The region of interest (ROI) was then delimited, including the interradicular region from the apical point closest to the mesial root to the apical point farthest from the distal root to the bone closest to the furcation. The ROI was delimited to the same region for all samples and included a set of 25 images each. To differentiate the cortical bone, trabecular bone, and bone marrow, the CTAn software adjusted the applicable threshold following the guidelines provided by the manufacturer, with a threshold (190–220) applied to the segmentation of the different scores of grey colour present in the image. Finally, the following parameters were measured: percent of bone volume (BV/TV), trabecular spacing (Tb.Sp), trabecular thickness (Tb.Th), and trabecular number (Tb.N) (Frazão et al. 2023; Matos‐Sousa et al. 2024).

The lesion volume was assessed by reconstructing the volume of interest (VOI) using CTAn software, which included the periodontal ligament space and the surrounding bone destruction. The examiner manually delineated the lesion area for each root of the first mandibular molar, starting at the mesial root and extending to the distal root. The initiation point for the VOI corresponded to the first coronal section where the mesial root was encased by the bone crest, and it continued distally until reaching the mandibular second molar (Frazão et al. 2023; Matos‐Sousa et al. 2024).

### 2.9 Statistical Analysis

The normality of the data was assessed with the Shapiro–Wilk test. Data analysis involved a one‐way ANOVA followed by Tukey's post hoc test. To examine group differences with two dependent variables, malondialdehyde (MDA) and reduced glutathione (GSH), a multivariate analysis of variance (MANOVA) was performed. Before conducting MANOVA, assumptions of multivariate normality (checked using the Shapiro–Wilk test) and homogeneity of covariance matrices were verified. A one‐way ANOVA followed by Tukey's post hoc test was applied to analyse the data. Data were expressed as mean ± standard error of the mean (SEM), with statistical significance set at *p* < 0.05. Statistical analyses were conducted using GraphPad Prism 9.0 (GraphPad Software, San Diego, CA, USA) and Jamovi version 2.3.38 (The jamovi project 2022).

## 3 Results

### 3.1 Validation of the Docking Protocol by Redocking of the Crystallographic Ligand

The superposition between the redocked and crystallographic ligand conformations revealed a high degree of spatial similarity, with a RMSD of 1.12 Å, indicating that the adopted protocol is robust and accurately reproduces the experimental orientation of the ligand within the binding pocket.

**FIGURE 4:**
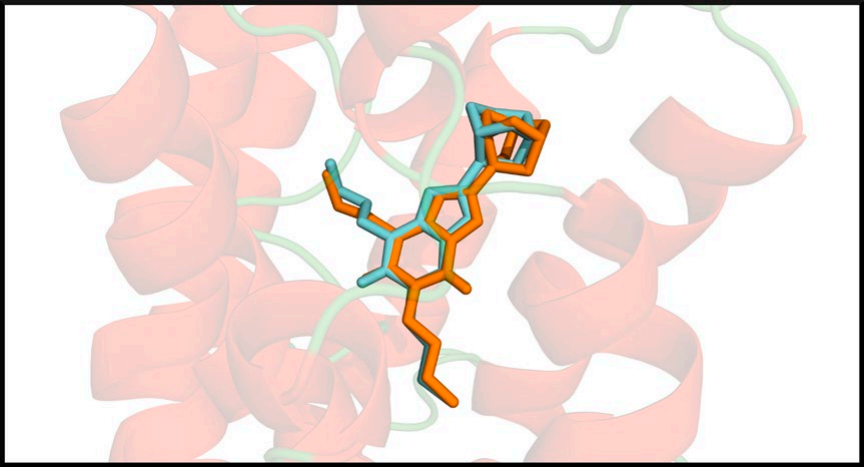
Superposition of the crystallographic ligand (cyan) and the redocked ligand (orange) within the binding pocket of the adenosine A_1_ receptor (PDB ID: 5N2S). The high degree of spatial overlap between both conformations demonstrates the accuracy and reproducibility of the docking protocol, confirming the reliability of the computational parameters used.

Figure 4 shows the superposition of the crystallographic ligand (cyan) and the redocked ligand (orange) within the binding site of the adenosine A_1_ receptor, highlighting the strong structural correlation between both conformations. This result validates the use of the docking protocol for subsequent simulations with the caffeine molecule, ensuring the reliability of the parameters employed in the molecular interaction analyses.

### 3.2 Predicted Binding Mode of Caffeine Within the Adenosine Receptor Active Site

**FIGURE 5:**
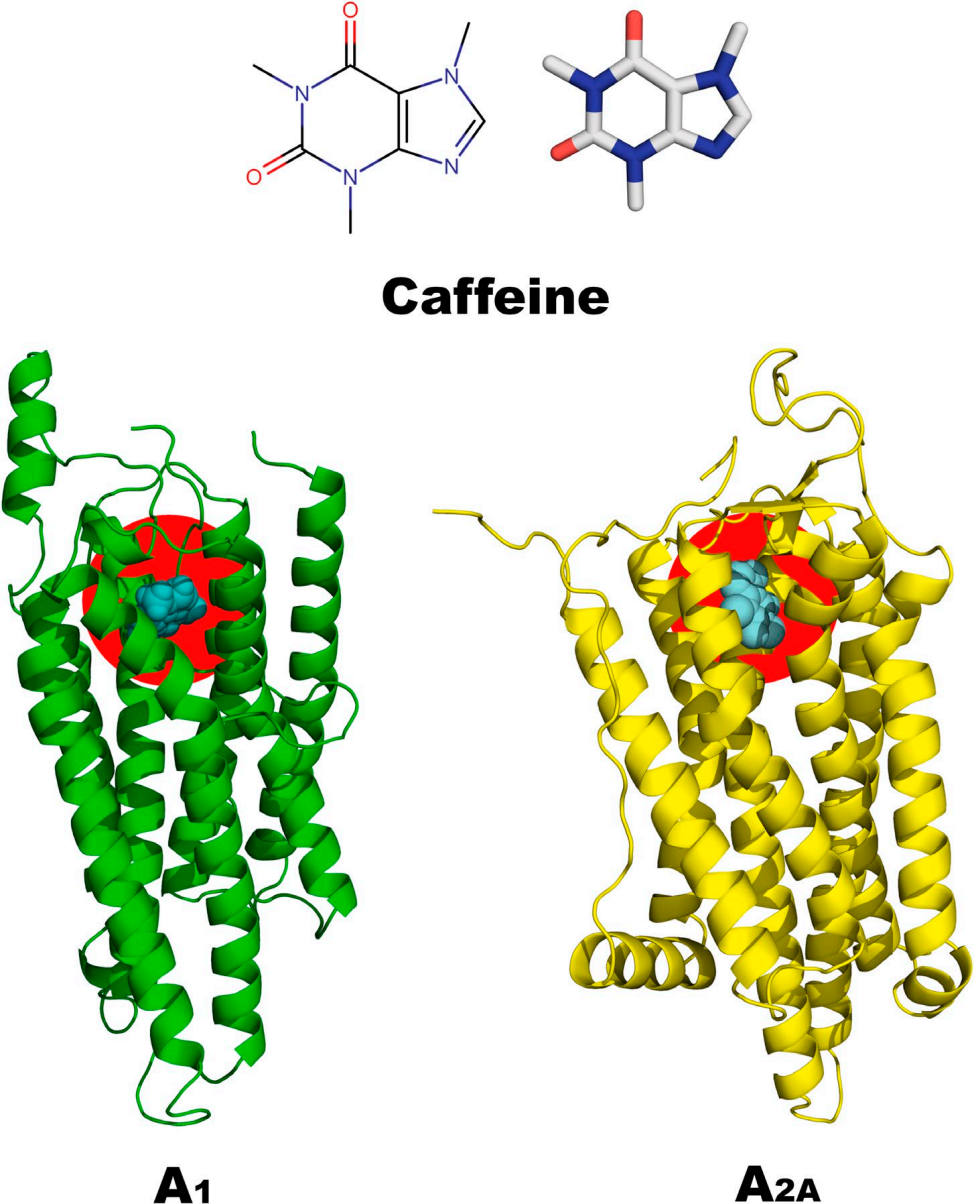
At the top of the figure, the two‐dimensional (2D) and three‐dimensional (3D) structures of caffeine are shown, with oxygen atoms highlighted in red and nitrogen atoms in blue. At the bottom, the three‐dimensional structures of the adenosine A_1_ (left) and A_2__A_ (right) receptors are displayed, with the proteins represented in ribbon format. The caffeine molecules are shown in blue, as spheres, positioned within the binding pocket, which is highlighted in red.

Figure 5 illustrates the binding cavity of caffeine within the adenosine A_1_ and A_2__A_ receptors, highlighting its position and orientation within the binding pocket.

The interaction energy of caffeine with the A_1_ receptor, calculated using Molegro Virtual Docker (version 5.5), was −71.3 kcal/mol. This value was not estimated for the A_2__A_ receptor, as the compound is already experimentally co‐crystallised within the binding pocket in the available crystal structure (PDB ID: 3RFM) (Doré et al. 2011).

**FIGURE 6:**
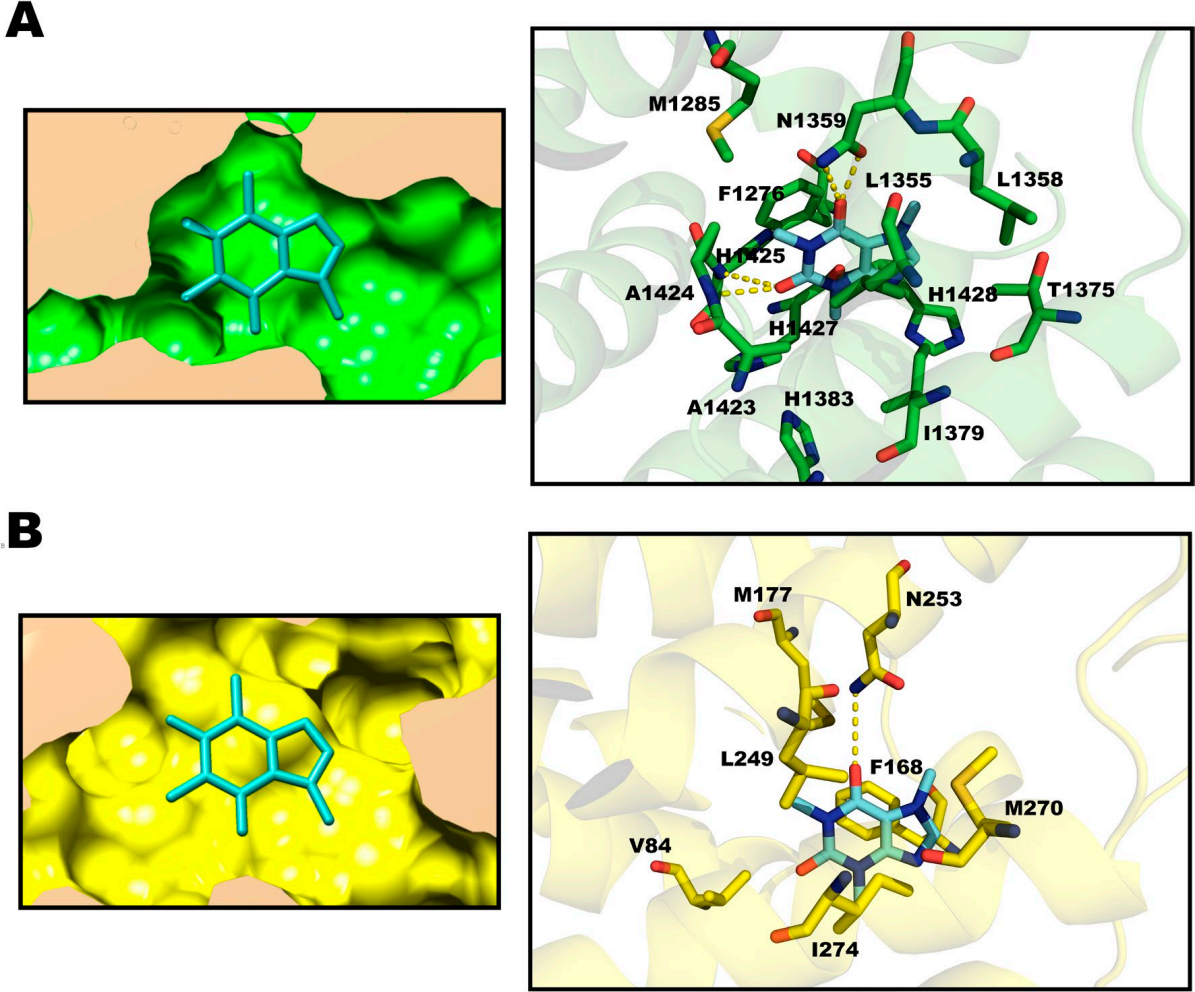
(A) On the left, caffeine is shown positioned within the binding site of the A_1_ adenosine receptor, with the binding pocket represented in surface mode. On the right, the same site is displayed with amino acid residues represented in stick format, allowing better visualisation of molecular interactions. (B) On the left, caffeine is inserted into the binding site in surface mode; on the right, the binding pocket is shown with amino acid residues in stick representation. In both panels, yellow dashed lines indicate hydrogen bonds. Oxygen atoms are shown in red, and nitrogen atoms in dark blue.

Figure 6 illustrates the interaction mode of caffeine with adenosine receptors, highlighting the amino acid residues involved in the formation of the drug–receptor complex.

Caffeine interactions within the adenosine A_1_ receptor binding pocket were predominantly hydrophobic, involving residues F1276, L1358, T1375, M1285, I1379, H1383, H1428, L1355, and H1427. In addition to these, four hydrogen bonds were identified—two with N1359, one with H1425, and one with A124. In the binding site of the A_2__A_ adenosine receptor, caffeine established hydrophobic interactions with residues V84, M177, L249, I274, M270, and F168, along with a single hydrogen bond with residue N253.

### 3.3 Antioxidant Activity of Caffeine

**FIGURE 7:**
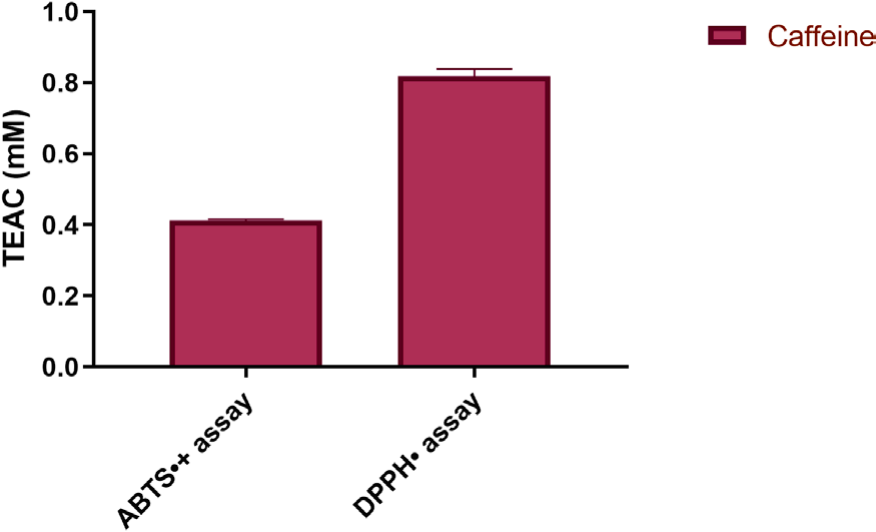
ABTS•+ and DPPH• radical scavenging assay and Trolox equivalent antioxidant capacity of Caffeine. Values are expressed as mean and standard deviation (*n* = 3) of Trolox equivalent antioxidant capacity.

The results of the ABTS•+ and DPPH• radical scavenging assays were presented as Trolox Equivalent Antioxidant Capacity (TEAC) using Trolox as a reference standard (Figure 7).

The DPPH• assay values were 0.818 ± 0.021 mM, and the DPPH• assay data show that caffeine is active in the presence of the DPPH• radical and has a good antioxidant capacity.

ABTS•+ values were 0.413 ± 0.003 mM. These results confirm that the antioxidant potentials of the samples were lower than the Trolox standard and lower when compared to the DPPH assay.

### 3.4 Caffeine Modulation of Oxidative Stress Triggered by Apical Periodontitis

**FIGURE 8:**
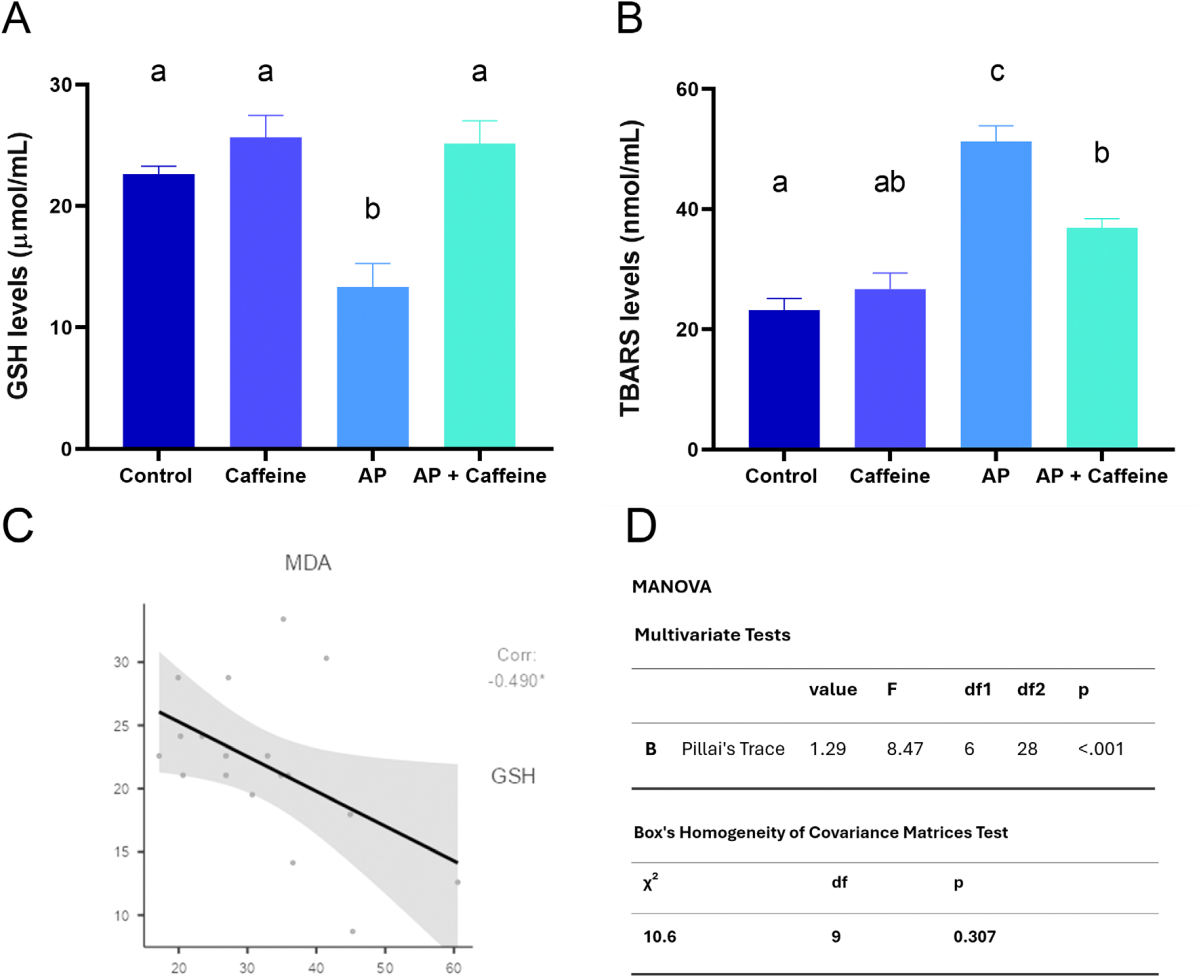
Biochemical analysis of plasma oxidative status of rats upon induction of apical periodontitis (AP) and caffeine administration (10 mg/kg/day) for 28 days. In (A) levels of reduced glutathione (GSH), and in (B) lipid peroxidation levels quantified as thiobarbituric acid reactive substances (TBARS). Different letters indicate statistical differences (*p* < 0.05) using an ANOVA test followed by Tukey's post hoc tests. Results are mean + standard error of the mean of 8 rats per group. (C) Presents a scatterplot with regression line illustrating the significant inverse correlation between MDA and GSH levels (Spearman's *ρ* = −0.490; *p* = 0.039). (D) Shows multivariate analysis (MANOVA) results showing significant group effects on combined MDA/GSH measures (Pillai's Trace), and validation of homogeneity of covariance matrices (Box's M).

AP animals displayed reduced GSH levels in blood samples (*p* = 0.0081, Figure 8), highlighting the oxidative impact of AP. Animals subject to AP and treated with moderate doses of caffeine (AP + caffeine group) exhibited higher GSH values than the AP group (*p* = 0.0007), reaching values similar to those of the control (*p* = 0.649) and caffeine groups (*p* = 0.996). This suggests that caffeine supplementation was able to mitigate the oxidative stress induced by AP.

The AP group displayed increased TBARS levels compared to the control group (*p* < 0.0001), indicating an increased lipid peroxidation reflecting the exacerbated oxidative stress caused by AP. The supplementation with caffeine (AP + caffeine group) lowered TBARS levels compared to the AP group (*p* = 0.0028), indicating a protective effect of caffeine on lipid peroxidation. Although the AP + caffeine group still presented higher values than the control group (*p* = 0.0045), no significant difference was found between the AP + caffeine and caffeine groups (*p* = 0.0624). Finally, the comparison between the control and caffeine groups revealed no statistically significant difference (*p* = 0.7181), suggesting that caffeine consumption did not affect TBARS levels in rats without AP.

Before conducting the correlation test and MANOVA, the Shapiro–Wilk test confirmed the multivariate normal distribution of the data (W = 0.935, *p* = 0.235). The correlation analysis showed a statistically significant inverse relationship between MDA and GSH levels (Spearman's *p* = −0.490, *p* = 0.039). The significant association (*p* < 0.05) suggests that higher oxidative stress, reflected by increased MDA levels, is associated with lower antioxidant capacity, as shown by decreased GSH levels. Additionally, multivariate analysis revealed a significant group effect on the combined dependent variables (Pillai's Trace = 1.29; *F*(6,28) = 8.47, *p* < 0.001), indicating substantial differences between groups in their multivariate profiles. Results of Box's M test show a non‐significant *p*‐value (*p* = 0.307), indicating that the assumption of equal variance–covariance matrices across groups was met (*χ* ^2^ = 10.6, df = 9), validating the use of MANOVA.

### 3.5 Caffeine Reduced the AP‐Associated Increase of Inflammatory Histopathology

**FIGURE 9:**
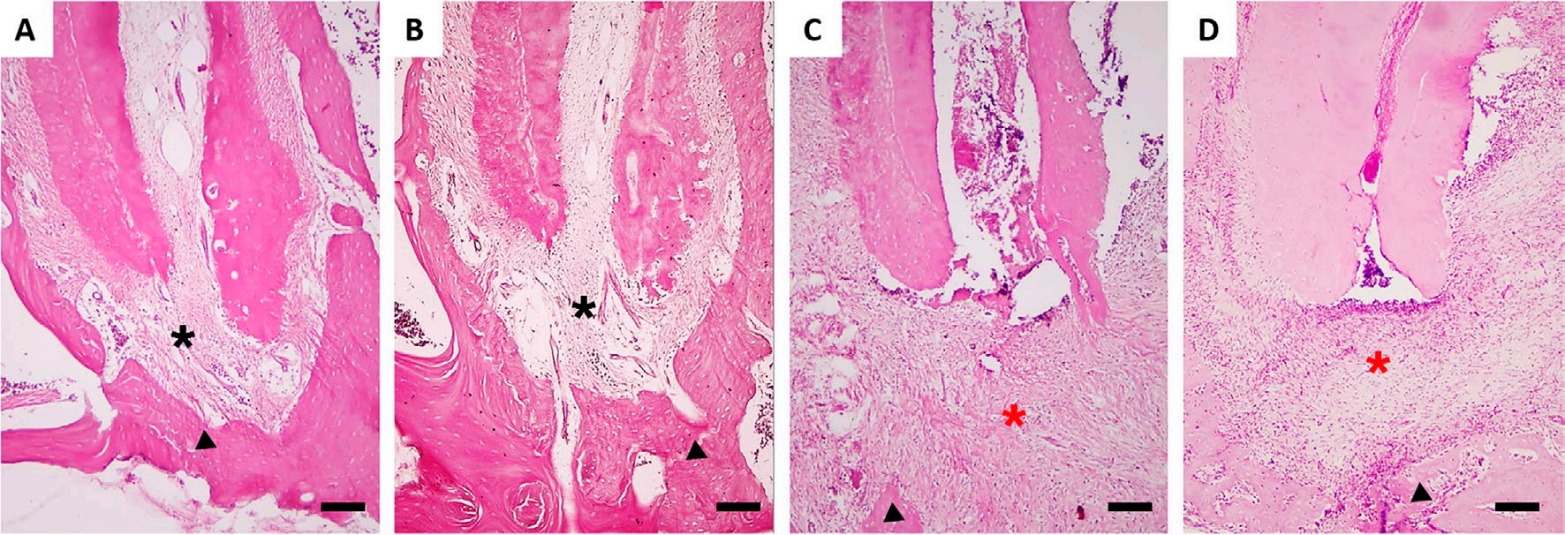
Representative photomicrographs of haematoxylin and eosin (HE) stained periapical tissues from experimental groups. Images show histopathological findings at the root apex in control (A), caffeine (B), apical periodontitis (C), and apical periodontitis + caffeine (D) groups. Black asterisks represent healthy periodontal ligament spaces. Red asterisks indicate areas of periapical lesion, characterised by inflammatory cell infiltration and bone resorption. Black triangles indicate areas of alveolar bone. It can be observed that the groups with apical periodontitis show reduced bone area, with only residual bone remaining, whereas the caffeine‐treated group exhibits a greater preserved bone area compared to the untreated group. Scale bar = 40 μm.

The histopathological analysis of the control group samples revealed well‐preserved connective and bone tissue (Figure 9). The underlying connective tissue was characterised by a dense arrangement of mature collagen fibres and a rich vascular supply. There was an absence of inflammatory cell infiltration, and the overall tissue architecture, including the periodontal ligament and alveolar bone crest (indicated by a black asterisk), was consistently maintained, indicative of healthy and physiological conditions. Similar to the control group, samples from the caffeine group demonstrated remarkable preservation of structural integrity. The periodontal ligament and alveolar bone architecture (indicated by a black asterisk) remained stable.

In contrast to the control and caffeine groups, samples from the AP group displayed extensive and severe histopathological alterations, indicative of a significant endodontic lesion. There was a pronounced disruption of the normal periapical tissue architecture. The connective tissue surrounding the tooth apex in the AP group exhibited intense inflammatory cell infiltration, mainly polymorphonuclear inflammatory cells. This severe periapical inflammatory process is consistent with an endodontic lesion, arising from the necrotic and infected pulp within the radicular canal. This severe inflammatory response was accompanied by significant destruction and disorganisation of collagen fibres, along with marked periapical bone resorption (indicated by a red asterisk) surrounding the apex of the tooth. The presence of polymorphonuclear inflammatory cells highlighted the impact of inflammation on the tissue microenvironment.

Samples from the AP + caffeine group revealed a notable amelioration of the inflammatory response compared to the AP group. While some degree of inflammatory cell infiltration was still evident, there was a discernible reduction in its intensity and extent. The inflammatory infiltrate appeared less dense and more focal, with a decreased proportion of polymorphonuclear cells. Furthermore, a relative preservation of collagen fibres was observed in certain areas, suggesting a protective or mitigating effect on connective tissue destruction. Although periapical bone resorption was still present (indicated by a red asterisk), its severity appeared to be attenuated in comparison to the AP group. These findings suggest that caffeine may exert a modulatory effect on the inflammatory processes associated with endodontic lesions, potentially contributing to a reduction in periapical tissue damage.

### 3.6 Caffeine Treated‐Group Showed More Collagen Area Than Non‐Treated

**FIGURE 10:**
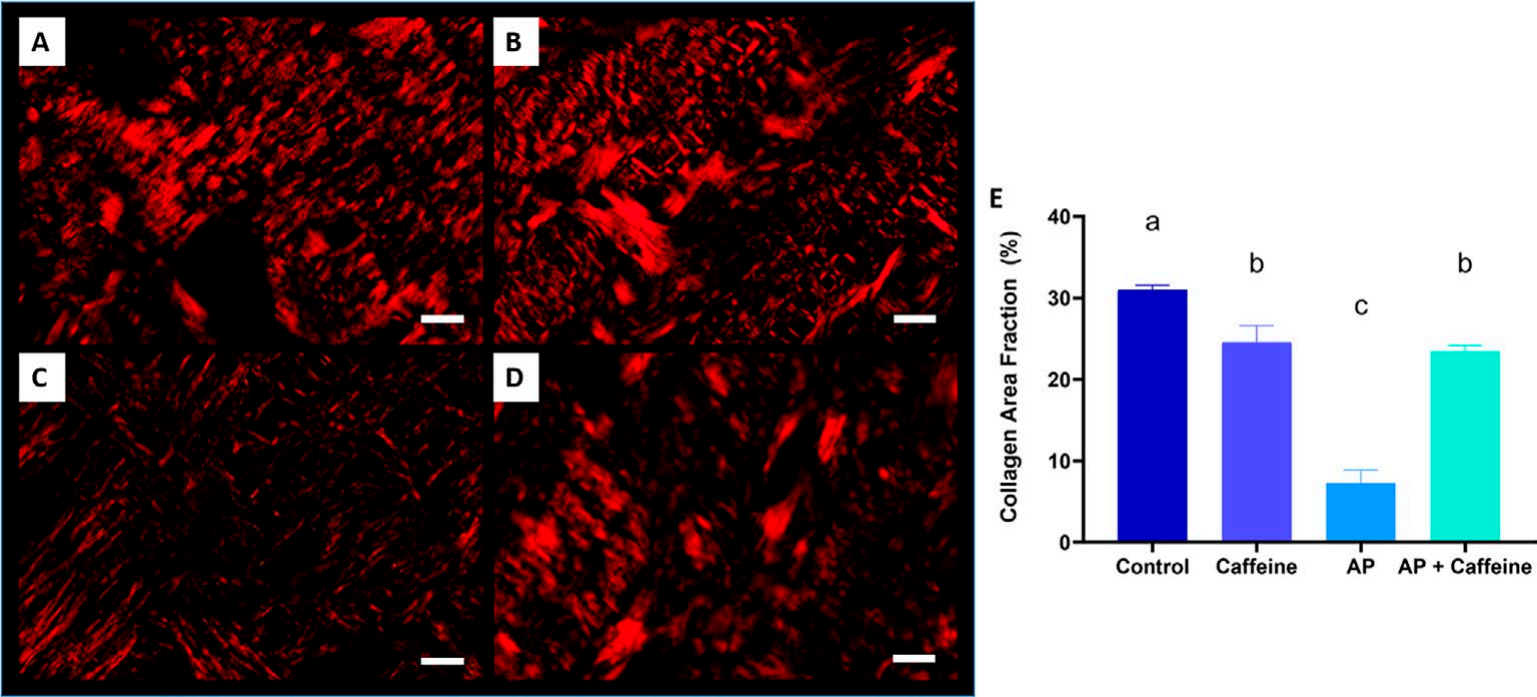
Representative PicroSirius red staining in control (A), caffeine (B), apical periodontitis (C), and apical periodontitis + caffeine (D). Panel (E) displays the percentage of bone collagen area (%), presented as mean + standard error of the mean of 8 rats per group. Different letters indicate statistical significance (*p* < 0.05) using a one‐way ANOVA followed by Tukey post hoc tests. Scale bars: 20 μm.

In the analysis of PicroSirius red staining, the AP group (7.28% ± 1.61; Figure 10) exhibited a reduction in the collagen area compared to the control group (31.0% ± 0.593; *p* < 0.0001), reflecting a destruction of the extracellular matrix. Although the AP + caffeine group (23.4% ± 0.714) did not reach control values (*p* = 0.0328 vs. control), the administration of caffeine in the AP rat model mitigated the loss of collagen staining induced by AP (*p* = 0.0106 vs. AP). The caffeine group (24.6% ± 2.06) did not differ significantly from the control group but exhibited higher collagen levels compared to the AP + caffeine group (*p* = 0.0183).

### 3.7 AP‐Induced Morphological and Microstructural Modifications of Apical Bone Are Attenuated by Moderate Caffeine Intake

**FIGURE 11:**
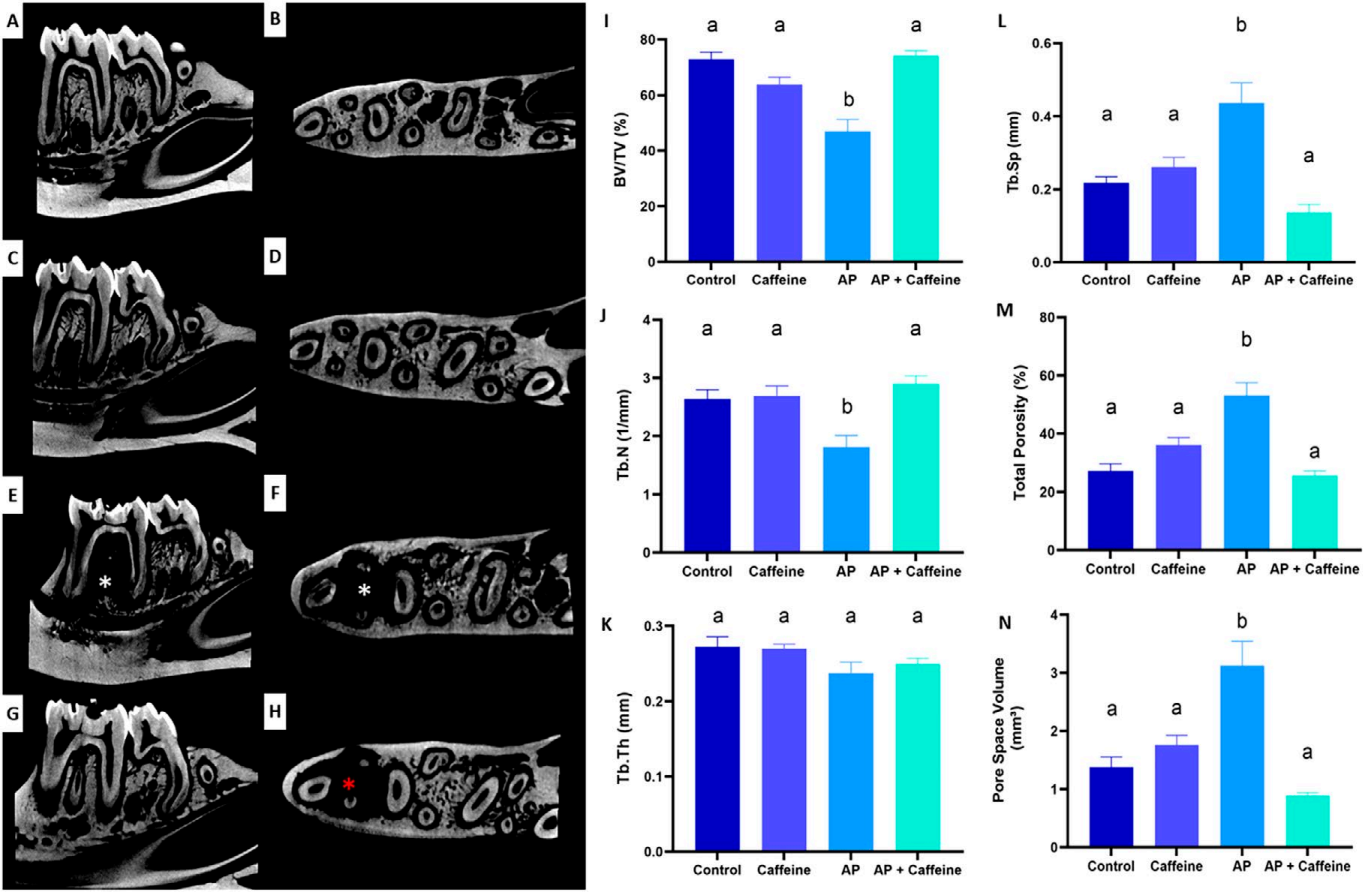
Representative microtomographic images of hemimandibles from the different experimental groups: control group (A, B), with sagittal (A), transverse (B); caffeine group (C, D), with sagittal (C), transverse (D); Apical Periodontitis (AP) group (E, F), with sagittal (E), transverse (F); and AP + caffeine group (G, H), with sagittal (G), transverse (H). The bar graphs summarise the microtomographic analysis. In (I), bone volume to total volume ratio (%); in (J), trabecular number (1/mm); in (K), trabecular thickness (mm); in (L), trabecular spacing (mm); in (M), Total Porosity (%); and (N), pore space volume (mm^3^). Different letters indicate statistically significant differences *p* < 0.05 using a One‐way ANOVA followed by Tukey's post hoc tests (*p* < 0.05). Results are mean ± standard error of 8 rats per group.

The analysis of BV/TV (Figure 11) showed that the BV/TV was significantly lower in the AP group compared to the control group (73% ± 2.6 vs. 47% ± 4.4; *p* = 0.0003). The AP + caffeine group exhibited mean BV/TV values (74% ± 1.7%) similar to the control group (*p* = 0.9879). Caffeine did not modify the BV/TV parameter in control rats (*p* = 0.2493).

Figure 11 shows that the Trabecular Number (Tb.N) was reduced in the AP group (1.8 ± 0.20 1/mm) compared to the control group (2.6 ± 0.16 1/mm; *p* = 0.0262). Caffeine administration (AP + caffeine group) preserved Tb.N compared to AP animals (2.9 ± 0.13 1/mm vs. 1.8 ± 0.20 1/mm; *p* = 0.0043), reaching similar scores to the control group (*p* = 0.6453).

In the Trabecular Thickness (Tb.Th) parameters, all groups evaluated exhibited similar scores. The control group had a mean Tb.Th of 0.27 ± 0.013 mm, while the AP group showed a mean of 0.24 ± 0.014 mm (*p* = 0.1711). The caffeine group exhibited a mean Tb.Th of 0.27 ± 0.0058 mm (*p* = 0.9988), and the AP + caffeine group showed a mean of 0.25 ± 0.0071 mm (*p* > 0.05).

Trabecular Spacing (Tb.Sp) values are shown in Figure 11. AP animals exhibited an increase of Tb.Sp compared to control rats (AP: 0.44 ± 0.055 mm; Control: 0.22 ± 0.017 mm; *p* = 0.0026). The AP + caffeine group displayed lower Tb.Sp values (0.14 ± 0.022 mm) compared to the AP group (*p* = 0.0004), reaching values similar to the control group (*p* = 0.3127).

In the percentage of total porosity parameter, the AP procedure increased total porosity (53% ± 4.4%) compared to the control group (27% ± 2.6%; *p* = 0.0003). Caffeine administration in AP animals (AP + caffeine group) prompted lower total porosity than the AP group (26% ± 1.6% vs. 53% ± 4.4%; *p* = 0.0002), with no significant difference compared to the control group (*p* = 0.9841).

The total Pore Space Volume was highest in the AP group (0.44 ± 0.055 mm^3^), being significantly greater than that in the control group (0.22 ± 0.017 mm^3^; *p* = 0.0020). The AP + caffeine group showed intermediate values (0.14 ± 0.022 mm^3^), significantly lower than those of the AP group (*p* = 0.0003) but not statistically different from the control group (*p* = 0.5327).

**FIGURE 12:**
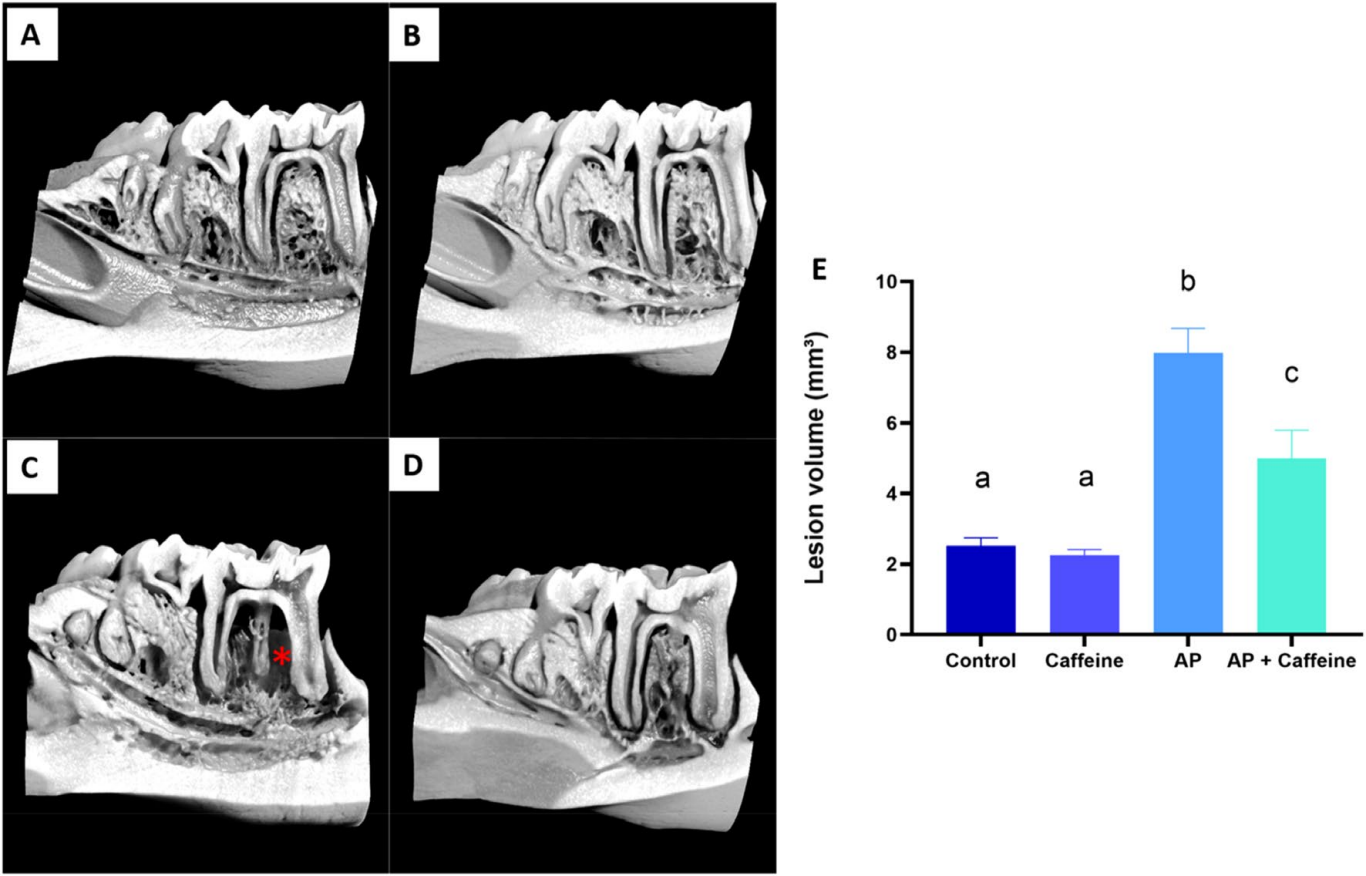
3D Reconstruction and lesion volume analysis of hemimandibles. Panels A–D depict representative 3D reconstructions of hemimandibles from the different experimental groups: (A) control, (B) caffeine, (C) Apical Periodontitis (AP), and (D) Apical Periodontitis with caffeine administration (AP + caffeine). The red asterisk highlights the periodontal ligament space or lesion region in the AP group. Panel E presents the lesion volume (mm^3^) for each group, expressed as mean ± standard error of the mean of 8 rats per group. Statistical significance (*p* < 0.05) is indicated by different letters *p* < 0.05 using a one‐way ANOVA followed by Tukey's post hoc tests.

The periodontal ligament volume varied significantly among the experimental groups (Figure 12). The AP group had the highest mean value (7.98 ± 0.694 mm^3^), significantly higher than the control group (2.51 ± 0.230 mm^3^, *p* < 0.0001) and the caffeine group (2.25 ± 0.163 mm^3^, *p* < 0.0001). Caffeine administration to rats modelling AP (AP + caffeine) resulted in a lower mean value of lesion volume (4.99 ± 0.798 mm^3^), showing a statistically significant difference compared to the AP group (*p* = 0.0106), suggesting that caffeine has a protective effect in the lesion progression.

## 4 Discussion

Our findings demonstrate that a moderate caffeine intake attenuates the progression of induced apical periodontitis in rats. In parallel, *in silico* analyses revealed that caffeine interacts with adenosine A_1_ and A_2__A_ receptors, providing mechanistic support for its anti‐inflammatory effects. Specifically, caffeine influenced the bone lesion volume, preserved bone quality and collagen integrity, and attenuated both the inflammatory response and oxidative stress associated with periapical tissue damage.

These beneficial effects were obtained using a moderate dose of caffeine (10 mg/kg) corresponding to the intake of 250 mg by humans, representing 2–3 cups of coffee (Fredholm et al. 1999), and contrast to the negative impact on AP of excessive caffeine doses of 100 mg/kg tested in a previous study (Dal‐Fabbro, Cosme‐Silva, de Rezen Silva Martins Oliveira, et al. 2021). This dose‐dependent health benefits versus adverse effects of caffeine, now concluded in the context of bone metabolism, is a general feature resulting from caffeine dose‐dependent pharmacodynamics (Lopes and Cunha 2024). In fact, caffeine acts on adenosine receptors at moderate doses and through different targets at higher doses (Fredholm et al. 1999). In contrast to the benefits now described to be afforded by moderate doses of caffeine, higher doses ranging from 25 to 100 mg/kg induce deleterious impacts on alveolar bone structure, particularly when combined with inflammatory or mechanical challenges (Bezerra et al. 2013; Yi et al. 2016; Dal‐Fabbro, Cosme‐Silva, Capalbo, et al. 2021). Moreover, even at moderate doses, caffeine can antagonise both adenosine A_1_ and A_2__A_ receptors, which play distinct roles in bone homeostasis: A_1_ receptors are expressed in both mature osteoblasts and osteoclasts, modulating resorption and formation, whereas A_2__A_ receptors are mainly present in osteoblasts and are involved in promoting bone formation (Mediero and Cronstein 2013). Consequently, the simultaneous antagonism of both receptors by caffeine may contribute to its divergent effects on bone remodelling, highly dependent on the dose administered and on the type of lesion. In this context, previous studies reported that even a moderate dose of caffeine (2–3 g/L) exacerbated alveolar bone loss associated with orthodontic movement in rats (Shirazi et al. 2017; Moreno et al. 2024), suggesting a harmful effect under these specific biomechanical conditions, in contrast to AP.

This discrepancy may stem from the fundamentally different pathological stimuli involved in orthodontic tooth movement, which is based on mechanical strain and compression triggering bone resorption via mechano‐transduction pathways (Li et al. 2021), whereas AP is primarily driven by microbial‐induced inflammation and oxidative stress (Wen et al. 2024). Notably, in another inflammatory model, caffeine counteracted ethanol‐induced bone damage (Maia et al. 2020), reinforcing its context‐dependent activity. Thus, while potentially harmful in mechanically loaded environments, moderate caffeine intake confers antioxidant and anti‐inflammatory protection in damage primarily mediated by microbial or toxicant stimuli, such as AP or ethanol binge‐like intake. Interestingly, from a translational perspective, a cross‐sectional study demonstrated that caffeine intake may act as a protective factor in the development of osteoporosis (Liu and Chai 2025). This finding corroborates the potentially beneficial effects of caffeine on bone health.

Docking results suggest that caffeine interacts with adenosine A_1_ and A_2__A_ receptors predominantly through hydrophobic interactions, complemented by hydrogen bonds with conserved residues in the active site, allowing the molecule to remain stably bound (Doré et al. 2011). In the A_1_ receptor, key interactions involve residues F1276, L1358, T1375, M1285, I1379, H1383, H1428, L1355, and H1427, in addition to four hydrogen bonds with N1359 and H1425. In the A_2__A_ receptor, beyond hydrophobic contacts with residues such as M177, F168, and I274, a prominent hydrogen bond is formed with N253—a key residue for accommodating xanthine‐type ligands within the binding pocket (Thomsen and Christensen 2006). The elucidation of these interactions provides molecular insights into the in vivo findings, particularly the reduction in inflammatory infiltrate, preservation of collagen integrity, and improvements in bone microarchitecture parameters.

In addition, corroborating the antioxidant potential of moderate doses of caffeine, our biochemical data showed that caffeine exhibits considerable antioxidant activity in scavenging radical species by the DPPH• and ABTS•+ assays. Interestingly, caffeine displayed higher antioxidant capacity in the DPPH• assay compared to the ABTS•+ assay. This variation might be attributed to differences in the reaction mechanisms or specific affinity of caffeine towards distinct radical species (Ősz et al. 2022; Vargas‐Pozada et al. 2023). From this perspective, these data confirm previous evidence about caffeine's antioxidant activity in moderate doses, suggesting a potential relevance in pathological conditions involving oxidative stress, such as periapical lesions.

Given that ROS are critically involved in the pathogenesis and progression of periapical lesions (Georgiou et al. 2021), the established antioxidant potential of caffeine justified the evaluation of its capacity to modulate oxidative stress and inflammation during the development of AP. Corroborating this scenario, our analysis demonstrated that AP triggers a significant redox imbalance, as evidenced by decreased levels of GSH and increased lipid peroxidation, measured via the TBARS method. GSH is a key non‐enzymatic intracellular antioxidant, and its depletion reflects intense oxidative activity and cellular stress. In our study, animals in the AP + Caffeine group showed a significant restoration of GSH levels, reaching values comparable to those observed in healthy animals. Similarly, TBARS levels were significantly reduced in the AP + Caffeine group compared to the AP group, indicating a protective role of caffeine against lipid peroxidation. Furthermore, the lack of significant differences between the control and caffeine groups in both GSH and TBARS confirms that moderate caffeine intake does not induce oxidative stress under physiological conditions. Collectively, these results suggest that caffeine not only possesses intrinsic antioxidant properties but also effectively modulates oxidative stress induced by AP in vivo. These findings are in agreement with previous studies that demonstrated similar outcomes using other antioxidants—such as N‐acetylcysteine (Şehirli et al. 2024), resveratrol (Dal‐Fabbro, Cosme‐Silva, de Rezen Silva Martins Oliveira, et al. 2021), and açaí (Moura et al. 2025)—capable of restoring redox balance and attenuating tissue damage in experimental models of AP and inflammatory bone loss.

In the current study, the periapical lesions were induced by pulp chamber exposure, allowing continuous contamination by oral microorganisms throughout the experimental period. We selected a 28‐day period for lesion development because it effectively represents the transition from acute inflammation, predominant within the first 14 days, to a chronic inflammatory condition thereafter (Minhoto et al. 2021; Okiji et al. 1994). Previous studies have described this well‐established experimental model for AP (Frazão et al. 2023; Matos‐Sousa et al. 2024). This timeframe simulates more closely the chronic nature of untreated human AP, providing a clinically relevant scenario to study the pathological mechanisms involved.

Our histopathological analysis revealed intense inflammatory infiltration, destruction of collagen fibres, and disruption of periodontal tissue architecture in the AP group, which were markedly attenuated in the AP + Caffeine group. These findings support the hypothesis that caffeine reduces tissue damage by limiting inflammatory cell recruitment and preserving extracellular matrix components. Notably, long‐term low to moderate doses of caffeine reduce chronic inflammation (reviewed in Dludla et al. 2023). Whereas A_2A_ receptors play a prime role in the onset of immune‐inflammatory responses (Sitkovsky et al. 2004), different adenosine receptors are engaged to control chronic inflammation (Haskó and Pacher 2008). This is in accordance with our previous observation that the selective blockade of A_2A_ receptors was not sufficient to reproduce the beneficial effect of caffeine in alveolar bone loss induced by ethanol binge drinking (Maia et al. 2020). Thus, while the present data posits the control of inflammation as a prime candidate mechanism to understand the impact of caffeine on AP, further studies should clarify the involvement of different adenosine receptors in this effect of caffeine.

In line with the histopathological findings, quantitative analysis of collagen area fraction confirmed the protective role of caffeine in preserving tissue structure. While animals in the AP group exhibited a dramatic reduction in collagen content, those treated with caffeine maintained significantly higher collagen levels, approximating those of the control group. This preservation of the extracellular matrix suggests that caffeine may help sustaining the structural and biomechanical integrity of periapical tissues, even under persistent inflammatory stimuli. These findings are aligned with the ability of caffeine and its main target—adenosine A_2A_ receptors—to promote tissue remodelling by affecting collagen production in different tissues (cf. Shaikh et al. 2016; Shan et al. 2022), to dampen the release of myeloperoxidase (Nobre Jr et al. 2010), to attenuate the impact of pro‐inflammatory cytokines (Simões et al. 2012), to control mitochondria‐derived formation of ROS (Castro et al. 2020), and oxidant‐mediated injury (Wang et al. 2022). In fact, like other natural antioxidants, low to moderate doses of caffeine may contribute to collagen preservation and extracellular matrix stability in mineralized tissues (Moreno et al. 2024).

Furthermore, micro‐CT analysis provided robust evidence of the structural preservation of alveolar bone in caffeine‐treated animals. AP led to bone loss, reflected by reductions in BV/TV, Tb.N, and increases in Tb.Sp, pore space volume, and total porosity. These findings may result from direct effects of adenosine receptors on bone formation and resorption (Mediero and Cronstein 2013) combined with the regulatory effect of caffeine on oxidative and inflammatory pathways that affect osteoclastogenesis and bone remodelling (Miao et al. 2024). Previous studies have demonstrated that natural antioxidants can mitigate bone microstructural deterioration in models of systemic inflammation or metabolic imbalance (Cao and Picklo 2014; Shen et al. 2011, 2009). Similarly, we previously reported that low to moderate caffeine was effective in preserving alveolar bone microarchitecture in adolescent rats subjected to ethanol‐induced systemic stress (Maia et al. 2020). These converging findings reinforce the potential of antioxidant agents, particularly caffeine, as modulators of bone integrity in inflammatory conditions such as AP. In clinical settings, the antioxidant and anti‐inflammatory properties of caffeine could serve as an adjuvant strategy to modulate the periapical environment, potentially accelerating tissue repair and bone healing following chemomechanical preparation. It is important to emphasise that caffeine is not supposed to substitute endodontic therapy; rather, its role would be to complement standard treatment by attenuating residual inflammation and oxidative stress, thereby creating more favourable conditions for periapical tissue regeneration.

Although this study provides significant insights into the systemic and osteoprotective effects of caffeine on the progression of AP, certain limitations deserve consideration. First, caffeine supplementation was administered without accompanying endodontic therapy, which limits the applicability of the findings to clinical scenarios where mechanical debridement is the standard treatment. Second, while micro‐CT and histopathological analyses demonstrated reduced lesion volumes, the molecular mechanisms underlying these effects, such as RANKL/OPG ratios, NF‐κB signalling, and TRAP activity, remain unexplored, resulting in gaps in understanding the molecular mechanism of caffeine's action in AP. Finally, the in silico approach consists of a prediction of molecular interactions that sometimes do not occur in vivo. Thus, additional molecular assays to explore the adenosine receptors density in the alveolar bone, as well as inflammatory biomarkers downstream signalling should be performed to elucidate caffeine's beneficial effects on AP. However, the present in silico results highlight an important pharmacological mechanism that may not be neglected.

## 5 Conclusion

This study demonstrates that moderate caffeine intake (10 mg/kg/day) significantly attenuates the progression of AP in rats by modulating oxidative stress, inflammatory responses, and bone remodelling. In vivo, caffeine reduced bone lesion volume, preserved trabecular microarchitecture, restored redox balance through intrinsic antioxidant activity, and maintained collagen integrity and tissue structure. Complementing these findings, *in silico* analyses indicated that caffeine interacts with adenosine A_1_ and A_2__A_ receptors, supporting its antagonistic activity and potential to modulate pro‐inflammatory signalling pathways. While clinical translation requires further investigation, particularly in combination with endodontic therapy, these results suggest that moderate caffeine intake could serve as a novel dietary modulator of oral bone homeostasis during inflammatory challenges. Future studies should further explore the molecular mechanisms to optimise therapeutic strategies for AP.

## Author Contributions

All authors contributed to the study conception and design. Material preparation and data collection were performed by Matheus Ferreira Lima Rodrigues and Deborah Ribeiro Frazão. Molecular docking was performed by Jorddy Neves Cruz, and the analysis and writing were performed by Jorddy Neves Cruz. Caffeine antioxidant activity analysis was performed by Jorddy Neves Cruz, and the analysis and writing were performed by Jorddy Neves Cruz. Histopathological analysis was performed by Matheus Ferreira Lima Rodrigues and Vinicius Ruan Neves dos Santos, and the analysis and writing were performed by Matheus Ferreira Lima Rodrigues. Computed X‐ray Microtomography (Micro‐CT) was performed by Vinicius Ruan Neves dos Santos and Fabrício Mezzomo Collares, and the analysis and writing were performed by Matheus Ferreira Lima Rodrigues, Felipe Oliveira Nunes, and Deborah Ribeiro Frazão. The discussion was written by Matheus Ferreira Lima Rodrigues, Deiweson Souza‐Monteiro, Thamires Campos Gomes, and João Daniel Mendonça de Moura. The manuscript was edited and reviewed by Cristiane do Socorro Ferraz Maia, Rodrigo A. Cunha, Rogerio de Castilho Jacinto, and Rafael Rodrigues Lima. All authors read and approved the final manuscript.

## Funding

This work was supported by the Conselho Nacional de Desenvolvimento Científico e Tecnológico (CNPq, Brazil) of the Brazilian Ministry of Science, Technology and Innovation through Researcher Productivity Grant (No. 307747/2025–5 to R.R.L.). Additional support was provided by the Coordenação de Aperfeiçoamento de Pessoal de Nível Superior (CAPES, Brazil), Ministry of Education, under Finance Code 001. R.R.L. also received funding from CNPq through research grants No. 408329/2022–0, No. 404431/2024–0, No. 400706/2024–5 and No. 409576/2025–5, as well as from the National Institute of Science and Technology in 3D Printing and Advanced Materials Applied to Human and Veterinary Health (INCT 3D‐Saúde, No. 406436/2022–3).

## Ethics Statement

This study was approved by the Animal Research Ethics Committee of the Federal University of Pará (number 2076280720). The study followed the ARRIVE 2.0 guidelines (du Sert et al. 2020), the NIH Guide for the Care and Use of Laboratory Animals (National Research Council (US) Committee for the Update of the Guide for the Care and Use of Laboratory Animals 2011), and the Preferred Reporting Items for Animal Studies in Endodontology (PRIASE) 2021 guidelines, including the PRIASE flowchart.

## Conflicts of Interest

The authors declare no conflicts of interest.

## Data Availability

Data sharing not applicable to this article as no datasets were generated or analysed during the current study.
